# American Surgery

**Published:** 1850-02

**Authors:** 


### AMERICAN SURGERY.

In the American edition of the London Lancet, for January, we find a report, of an introductory lecture delivered at the London Hospital, by Dr. Letheby, in which that gentleman uses the following language, in reference to the labors of American surgeons: "our brethren upon the new continent had this year published their first annual report on the condition of the medical sciences in that country; and while he agreed with the reporter when he states that the great *forte* of American medical scholarship has hitherto consisted in editing the works of British authors, that the fairest fruits of British genius and research are shaken into the lap of the American student, and that the creative energy of that country shall manifest itself in generating a race of *curculios*, to revel in voracious indolence on the products of a foreign soil, yet he could not place a like amount of faith in many of the exaggerated statements which are there put forth, and he illustrated this by a reference to American lithotomy practice, which showed an average mortality of only three per cent., that of British practice being about fifty !"

Passing over the ill-tempered and unjust allusions to American medical literature, with the single remark that we know of but few English works, of the best class, that have been edited in this country, which were not materially improved by the American editor—Cooper's Surgical Dictionary, for example—we take up the gratuitous and unfounded imputation on the veracity of American surgeons, in reference to lithotomy practice. It is hard, we admit, for a British metropolitan surgeon to understand how an American surgeon can be much more successful than one of the parent stock, but surely some better way might be found for examining the matter, than an unfounded assault upon the veracity, honor, and integrity of a class of men who have no superiors on the earth for truthfulness and uprightness. Why should American surgeons be any more likely to be guilty of dishonorable and degrading practices, in their profession than English surgeons? Why should it be imagined that the Warrens, Motts, and Gibsons of the United States are more prone to exaggeration, or falsehood, than the Stanleys, and Lethebys of London? The American public would speedily understand the resort to improper arts, and would administer a rebuke to such conduct that would not be forgotten. We should think that Dr. Letheby would have been more honestly and professionally employed, if he had endeavored to ascertain the causes of the greater success of American surgeons, in lithotomy, instead of calumniating a set of men who are quite as incapable of a dishonorable deed as he is. The fact which he endeavors to impugn is fixed too firmly to be shaken by sneers and inuendoes.—Death from lithotomy is very rare in this country, and scarcely amounts to three per cent. The particular surgeon at whom Dr. Letheby aims his shafts, has unquestionably been the most successful surgeon of the day, in lithotomy, and deserves admiration, rather than calumny. He practices in a community, where every case in his hands is known to numbers of citizens, and it would be impossible for him to exaggerate his success, without detection. He has enemies enough to emblazon his failures, if there were many to point at. We lived in the community, where he practices, for a quarter of a century, and during the time he was occupied in a large proportion of his operation, and we feel sure that we knew of every case he operated upon, and of every loss. As far as our word can corroborate the statements of that distinguished surgeon, we bear testimony to the fact, that it is his great success that has made the average per cent. of loss, in American surgery, so very diminutive, when compared with British surgery.

French surgery, true to the instincts of professional advancement, when it heard of Chesselden's success in lithotomy, sent Morand over to England, under the authority of the French Royal Academy of Sciences, to learn from Chesselden his method of operating. This was a much more honorable and *improving* course than the one Dr. Letheby has preferred for his action, and he might improve his surgery by following the example set him by the French Academy. A little investigation would teach Dr. Letheby, that British surgery has lost ground since the days of Chesselden. Dr. Letheby admits that there is a loss of fifty per cent., in British lithotomy, but it was not so in the hands of Chesselden, after he improved himself into his third method of operation.—He lost four out of ten patients, under his first method, and determined to improve himself, and by the third method he adopted, he saved fifty patients out of fifty-two, in St. Thomas's Hospital. Did Chesselden exaggerate, or has British lithotomy retrograded? When Sharpe was in Paris, in 1702, Frère Jacques had cut thirty-eight patients, without losing one. In view of such facts, where is the sense or propriety of Dr. Letheby's sneer at American surgery? Why not inquire why American surgery is so very scccessful?

